# Ethyl Lauroyl Arginate-Integrated Lipid Nanoparticles as a Multifunctional Non-Antibiotic Platform: Physicochemical Stability, Follicular Penetration, and Broad-Spectrum Activity Against Canine Skin Pathogens

**DOI:** 10.3390/pharmaceutics18091165

**Published:** 2026-09-16

**Authors:** Kittipat Supchukun, Teerapong Yata, Jakarwan Yostawonkul, Benchaphorn Limcharoen, Sayamon Srisuwatanasagul

**Affiliations:** 1 International Graduate Course of Veterinary Science and Technology (VST), Faculty of Veterinary Science, Chulalongkorn University, Bangkok 10330, Thailand; 6478310731@student.chula.ac.th; 2 Premier Innova Company Limited, Bangkok 10250, Thailand; 3 National Nanotechnology Center, National Science and Technology Development Agency, Thailand Science Park, Klong Luang 12120, Thailand; 4 Department of Anatomy, Faculty of Veterinary Science, Chulalongkorn University, Bangkok 10330, Thailand; benchaphorn.l@chula.ac.th; 5 Center of Excellence in Materials and Biointerfaces, Chulalongkorn University, Pathumwan, Bangkok 10330, Thailand

**Keywords:** ethyl lauroyl arginate hydrochloride (LAE), lipid-based nanoparticles, canine superficial pyoderma, topical antimicrobial delivery

## Abstract

**Background/Objectives:** Canine superficial pyoderma is one of the most common dermatological diseases in dogs and is increasingly complicated by antimicrobial resistance. This study aimed to develop ethyl lauroyl arginate-integrated lipid-based nanoparticles (LAE-LBNs) as a non-antibiotic topical delivery platform and to evaluate their physicochemical stability, skin localization, and antimicrobial activity against clinically relevant canine skin pathogens. **Methods:** LAE-LBNs were prepared using high-shear homogenization followed by probe ultrasonication and characterized for particle size, polydispersity index, zeta potential, morphology, encapsulation efficiency, and storage stability. Antimicrobial activity was evaluated against methicillin-susceptible *Staphylococcus pseudintermedius* (MSSP), methicillin-resistant *S. pseudintermedius* (MRSP), and *Malassezia pachydermatis*. The ex vivo distribution of Nile Red-labeled formulations was assessed in porcine ear skin using fluorescence microscopy. **Results:** The optimized 5% LAE-LBN formulation exhibited a relatively small particle size, narrow size distribution, strongly positive surface charge (+46.49 mV), encapsulation efficiency exceeding 99%, and favorable physicochemical stability over two months. Fluorescence associated with the labeled LAE-LBN formulation was observed in the stratum corneum, viable epidermis, and hair follicles, whereas the aqueous control formulation was predominantly confined to the stratum corneum. LAE-LBNs exhibited bactericidal and fungicidal activity against MSSP, MRSP, and *M. pachydermatis*, with an MBC or MFC of 12.2 µg/mL. These endpoints were identical to those obtained for aqueous LAE, indicating that incorporation into the lipid-based nanocarrier preserved the antimicrobial activity of LAE. **Conclusions:** LAE-LBNs combined favorable physicochemical properties, follicular localization, and activity against bacterial and fungal canine skin pathogens. These findings support their further investigation as a non-antibiotic topical delivery platform for canine superficial pyoderma.

## 1. Introduction

Canine superficial pyoderma (CSP) is among the most common infectious skin diseases encountered in veterinary practice and remains a leading cause of dermatological consultations in dogs [1]. Predominantly caused by *Staphylococcus pseudintermedius*, CSP is characterized by recurrent superficial bacterial infections that compromise animal welfare, increase treatment costs, and frequently require prolonged antimicrobial therapy [2].

In addition to bacterial pathogens, the lipophilic yeast *Malassezia pachydermatis* commonly contributes to canine skin disorders and frequently coexists with bacterial infections, exacerbating chronic inflammation, pruritus, and recurrent disease [3,4]. The increasing prevalence of multidrug-resistant and methicillin-resistant *S. pseudintermedius* (MRSP) has become a major therapeutic challenge, highlighting the need for alternative non-antibiotic treatment strategies [5,6]. The currently available topical formulations for these infections, including shampoos, sprays, mousses, wipes, and antiseptic solutions, often suffer from limited residence time on the skin because of grooming behavior, bathing, mechanical abrasion, and environmental exposure [3,4].

Moreover, the barrier properties of the stratum corneum restrict penetration of active compounds into deeper epidermal layers and pilosebaceous units [7,8]. Because hair follicles are major microbial reservoirs and potential sites of persistent infection [9], improving drug retention and delivery within these structures may enhance localized antimicrobial efficacy while reducing the need for frequent reapplication [3,4,10,11,12]. These limitations highlight the need for advanced topical delivery systems capable of prolonging skin residence, promoting follicular localization, and providing sustained antimicrobial activity [13,14].

Ethyl lauroyl arginate, also known as Ethyl Nα-lauroyl-L-arginate hydrochloride (LAE), is a cationic surfactant derived from L-arginine and lauric acid that has attracted increasing attention as a biodegradable, non-antibiotic antimicrobial agent because of its broad-spectrum activity and favorable safety profile [15]. Structurally, LAE contains a positively charged guanidinium group that interacts electrostatically with negatively charged microbial membranes, leading to membrane disruption, leakage of intracellular contents, and microbial cell death [16,17,18].

In addition, several studies have suggested that LAE may promote the generation of intracellular reactive oxygen species, thereby further contributing to antimicrobial activity [19]. These combined mechanisms confer potent activity against both Gram-positive and Gram-negative bacteria [18,20,21,22], and fungal species such as *Candida albicans* and other clinically relevant yeasts [17,23], making LAE particularly attractive for veterinary dermatology where mixed bacterial and fungal infections are frequently encountered [17,20,23]. As an amphiphilic molecule, LAE reduces surface tension, stabilizes emulsions, and promotes self-assembly above its critical micelle concentration [17,24]. Consequently, LAE may serve not only as the active antimicrobial agent but also as a cationic surface modifier and interfacial stabilizer within nanostructured delivery systems. Recent studies have further demonstrated that nanoencapsulation preserves the potent anti-biofilm activity of LAE while maintaining excellent biocompatibility and minimal cytotoxicity [25], supporting its suitability for advanced topical antimicrobial formulations. Nevertheless, the potential of LAE as a topical nanomedicine, especially for veterinary skin infections, has not yet been explored.

Lipid-based nanoparticles (LBNs), including nanostructured lipid carriers, have emerged as promising vehicles for topical drug delivery because they improve formulation stability, prolong skin residence, enable controlled drug release, and facilitate localization within hair follicles [8,26].

Unlike inorganic antimicrobial nanoparticles, such as silver nanoparticles, whose long-term applications remain limited by concerns regarding cytotoxicity and environmental persistence [27,28], lipid-based systems are generally regarded as biocompatible and biodegradable. Collectively, these properties may improve local antimicrobial retention at sites of microbial colonization while reducing reliance on conventional antibiotics.

Therefore, this study aimed to develop and optimize LAE-integrated lipid-based nanoparticles (LAE-LBNs) as a non-antibiotic topical antimicrobial platform for canine microbial skin infections. We hypothesized that incorporating LAE into lipid-based nanoparticles would produce a physicochemically stable, positively charged formulation that retains the antimicrobial activity of LAE and promotes its cutaneous and follicular localization. These properties were evaluated against clinically relevant canine skin pathogens and in an ex vivo porcine skin model used as a mammalian skin surrogate for topical-delivery assessment.

## 2. Materials and Methods

### 2.1. Chemicals

Ethyl Nα-lauroyl-L-arginate hydrochloride (LAE; CAS No. 60372-77-2; molecular formula C_20_H_41_ClN_4_O_3_; molecular weight 421.02 g/mol; National Center for Genetic Engineering and Biotechnology (BIOTEC), Nonthaburi, Thailand), hereafter referred to as LAE, was used without further purification. Glycerol (Cat. No. ET82132/0020/8S06, ≥99.5%; Chem Plus Trading, Bangkok, Thailand), medium-chain triglyceride (MCT) oil, polyethylene glycol (PEG) 100, polyethylene glycol (PEG) 4000, cholesterol, sorbitan monooleate (Span 80), and cetyl alcohol (Myskin Recipe Co., Ltd., Bangkok, Thailand) were used without further purification. All other chemicals and reagents were of analytical grade.

### 2.2. Microorganisms

Clinical isolates of *Malassezia pachydermatis* (M293/2025), methicillin-resistant *Staphylococcus pseudintermedius* (MRSP; M673/2025), and methicillin-susceptible *Staphylococcus pseudintermedius* (MSSP; M652/2025) were originally isolated from canine clinical samples submitted by small animal hospitals to the Diagnostic Laboratory, Department of Veterinary Microbiology, Faculty of Veterinary Science, Chulalongkorn University, Bangkok, Thailand. The use of these clinical isolates in the present study was approved by the Institutional Biosafety Committee of the Faculty of Veterinary Science, Chulalongkorn University, Bangkok, Thailand (Approval number IBC2531011).

### 2.3. Preparation of LAE-Integrated Lipid-Based Nanoparticles

LBNs containing different concentrations of LAE were prepared using a combination of high-shear homogenization and ultrasonication. Four formulations were prepared, consisting of a blank nanocarrier (0% LAE) and LAE-integrated formulations containing 1%, 5%, and 10% (*w*/*v*) LAE.

The oil phase consisted of MCT oil (10% *w*/*v*), PEG-100 (1% *w*/*v*), Span 80 (5% *w*/*v*), cetyl alcohol (1% *w*/*v*), and cholesterol (1% *w*/*v*). This mixture was heated to 70–75 °C under continuous stirring until a homogeneous phase was obtained. The aqueous phase was prepared by dissolving LAE in purified water (q.s. to 100% *w*/*v*) at 60–70 °C under constant stirring until a clear solution was obtained. PEG-4000 (0.5% *w*/*v*) was then added and stirred until completely dissolved. The aqueous phase was gradually added to the oil phase while maintaining the same temperature and mixed thoroughly. The resulting emulsion was subjected to high-shear homogenization at 10,000 rpm for 10 min, followed by probe ultrasonication at 40% amplitude for 10 min using 30-s pulses with 10-s intervals to produce LAE-integrated lipid-based nanoparticles. The dispersion was then cooled to room temperature. The same preparation procedure was applied to all formulations, with the LAE concentration adjusted to obtain final concentrations of 0%, 1%, 5%, or 10% (*w*/*v*).

For fluorescence imaging studies, Nile Red-labelled LAE-LBNs were prepared by incorporating Nile Red into the oil phase prior to emulsification. As a hydrophobic dye, Nile Red preferentially partitions into the lipid core of the nanoparticles during self-assembly, providing stable fluorescent labeling without altering the preparation procedure.

### 2.4. Physicochemical Characterization of LAE-LBNs 

The physicochemical stability of all formulations was evaluated during storage at 4 °C (refrigerated storage), 25 °C (ambient storage), and 45 °C (accelerated thermal stress condition) for up to 2 months. Samples were collected after 1 and 2 months, and changes in hydrodynamic diameter, polydispersity index (PDI), and zeta potential (particle surface charge) were determined using dynamic light scattering (DLS) with a Zetasizer Nano ZS (Malvern Instruments, Worcestershire, UK). Before measurement, the nanoparticle dispersions were diluted 1:50 with deionized water and analyzed at 25 °C. All measurements were performed in triplicate.

As part of routine quality-control characterization, the initial pH of the undiluted formulations and LAE-Aq was measured at room temperature using a calibrated pH meter (FiveEasy pH meter F20, Mettler Toledo, Greifensee, Switzerland). The electrode was rinsed with deionized water between samples, and pH measurements were performed in triplicate.

### 2.5. Morphological Characterization of LAE-LBNs

Following selection of the optimal formulation (5% LAE-formulation), the particle size and morphology of LAE-LBNs were characterized using transmission electron microscopy (TEM; JEM-2100, JEOL Ltd., Tokyo, Japan) operated at an accelerating voltage of 80 kV. The nanoparticle dispersion was diluted 1:50 with deionized water (pH 7.0), deposited onto carbon-coated copper grids, and air-dried. The grids were negatively stained with UranyLess staining solution (Fisher Scientific, Waltham, MA, USA) for 2 min, rinsed gently with deionized water, air-dried, and subsequently examined by TEM. Particle diameters were determined from calibrated TEM micrographs using the automated measurement function integrated into the TEM imaging software (iTEM; JEM-2100, JEOL Ltd., Tokyo, Japan).

### 2.6. Fourier-Transform Infrared Spectroscopy (FTIR)

Fourier transform infrared (FT-IR) spectroscopy was performed to characterize aqueous LAE (LAE-Aq; containing the same LAE concentration as the selected nanoformulation), the blank nanocarrier, and the selected LAE-integrated lipid-based nanoparticles (LAE-LBNs) using an ATR-FTIR spectrometer (INVENIO S/Lumos II, Bruker Optik GmbH, Ettlingen, Germany). Spectra were recorded over the range of 4000–400 cm^−1^ at a spectral resolution of 4 cm^−1^ with 64 scans per spectrum.

### 2.7. Encapsulation Efficiency of LAE-LBNs

The encapsulation efficiency (EE) of LAE in LAE-LBNs was determined using a regenerated cellulose centrifugal filter unit with a molecular weight cut-off (MWCO) of 30 kDa (Amicon Ultra-15, Merck Millipore Ltd., Darmstadt, Germany) following previously described methods [29,30].

Briefly, 1.5 mL of the LAE-LBN dispersion was transferred into the centrifugal filter unit and centrifuged according to the manufacturer’s instructions. The filtrate containing free (unencapsulated) LAE and supernatant were collected, passed through a 0.22 μm nylon syringe filter, and analyzed using a high-performance liquid chromatography (HPLC) system (Waters e2695, Waters Corporation, Singapore) equipped with a photodiode array detector (Waters 2998 Photodiode array detector, Waters Corporation, Singapore). Chromatographic separation was performed using a Symmetry C18 column (150 × 3.9 mm, 5 μm) using a mobile phase consisting of acetonitrile and water (50:50, *v*/*v*) containing 0.1% trifluoroacetic acid. The flow rate, injection volume, and detection wavelength were set to 1.0 mL/min, 20 μL, and 215 nm, respectively, according to the analytical method described by the Joint FAO/WHO Expert Committee on Food Additives [31]. The encapsulation efficiency was calculated using the following equation:

(1) EE (%) = [(Ci − Cf)/Ci] × 100

where Ci is the initial concentration of LAE used for nanoparticle preparation, and Cf is the concentration of free (unencapsulated) LAE in the filtrate.

### 2.8. Dialysis-Based Assessment of Receptor-Phase LAE Availability

The time-dependent transfer of LAE from LAE-LBNs and aqueous LAE (LAE-Aq) into the receptor medium was evaluated using a dialysis bag method. Briefly, 2 mL of LAE-LBNs or LAE-Aq was transferred into dialysis tubing (molecular weight cut-off: 3.5 kDa; Merck MilliporeBurlington, MA, USA) and immersed in 30 mL of receptor medium consisting of phosphate-buffered saline (PBS, pH 6.5) and ethanol (95:5, *v*/*v*). The experiment was conducted at 36 °C with continuous shaking at 200 rpm.

Sink conditions were assessed by comparing the maximum theoretical LAE concentration in the receptor compartment with the reported aqueous solubility of LAE. Each dialysis bag contained 100 mg of LAE; therefore, complete transfer into the 30 mL receptor medium would result in a maximum theoretical concentration of 3.33 mg/mL. This concentration was more than 74-fold lower than the reported aqueous solubility of LAE (>247 mg/mL at 20 °C) [32].

At predetermined time points (0, 0.5, 1, 3, 6, 12, 24, 48, and 72 h), 1 mL of the receptor medium was withdrawn and immediately replaced with an equal volume of fresh medium to maintain a constant receptor volume. The collected samples were filtered through a 0.22 μm nylon syringe filter and analyzed by HPLC as described in Section 2.7. The measured concentrations were used to describe the time-dependent appearance of LAE in the receptor compartment under the specified dialysis conditions. Because LAE recovery following contact with the dialysis membrane and nylon syringe filter was not independently evaluated, the measured concentrations were not interpreted as absolute drug-release values.

### 2.9. Evaluation of Antimicrobial Activity 

The antimicrobial activity of LAE-LBNs, LAE-Aq, and the blank nanoparticle carrier was evaluated against the clinical isolates described in Section 2.2. Species identification of all isolates was confirmed by matrix-assisted laser desorption/ionization time-of-flight mass spectrometry (MALDI-TOF MS) (Bruker Daltonics GmbH, Bremen, Germany).

Briefly, two-fold serial dilutions (1:2–1:4096) of each formulation were prepared in phosphate-buffered saline (PBS) and mixed with microbial suspensions (approximately 1 × 10^6^ CFU/mL). The mixtures were incubated at room temperature for 1 h for MRSP and MSSP and *Malassezia pachydermatis*. Following incubation, 10 μL aliquots were spread onto 5% sheep blood agar for *S. pseudintermedius* or Sabouraud dextrose agar (SDA) for *M. pachydermatis*. Plates were incubated at 37 °C for 20–24 h (*S. pseudintermedius*) or 72 h (*M. pachydermatis*) [33].

Positive and negative controls consisted of microbial suspension without treatment or formulation, and PBS without microorganisms, respectively. Colony-forming units (CFU) were enumerated in triplicate. The minimum bactericidal concentration (MBC) and minimum fungicidal concentration (MFC) were defined as the lowest concentration at which no visible colony growth was observed.

### 2.10. Ex Vivo Porcine Skin Model for Follicular Localization of LAE-LBNs

Fresh porcine ears without abnormal skin lesions were obtained from the necropsy unit, Faculty of Veterinary Science, Chulalongkorn University (Animal Use Protocol 2631019, Chulalongkorn University Animal Care and Use Committee). The ears were rinsed thoroughly with tap water, and excess hair was carefully trimmed using scissors. Nile red-labeled LAE-LBNs, as described in Section 2.3, were topically applied at a dose of 20 µL/cm^2^ to the skin and gently massaged into the skin using circular motions [34,35]. The treated skin was maintained at room temperature for 1 h to allow nanoparticle penetration. Following incubation, full-thickness skin biopsies (5 × 5 mm) were excised using a surgical blade, rapidly frozen using Freezing medium spray (CryoSpray, Bio-Optica, Milan, Italy), and sectioned into 10 μm-thick cryosections with a cryostat (Leica CM1950, Leica Microsystems, Wetzlar, Germany). A minimum of 10 hair follicles was evaluated for each skin sample. The localization of LAE-LBNs within the hair follicles was examined using an inverted fluorescence microscope (Zeiss Apotome 3, Carl Zeiss, Oberkochen, Germany) without further tissue processing. Nile red fluorescence (λex/em = 553/637 nm) was imaged using a 10× objective, and the follicular localization depth was quantified using ZEN software 3.10 (Carl Zeiss, Oberkochen, Germany).

## 3. Results

### 3.1. Physicochemical Properties of LAE-LBNs

**Figure 1:**
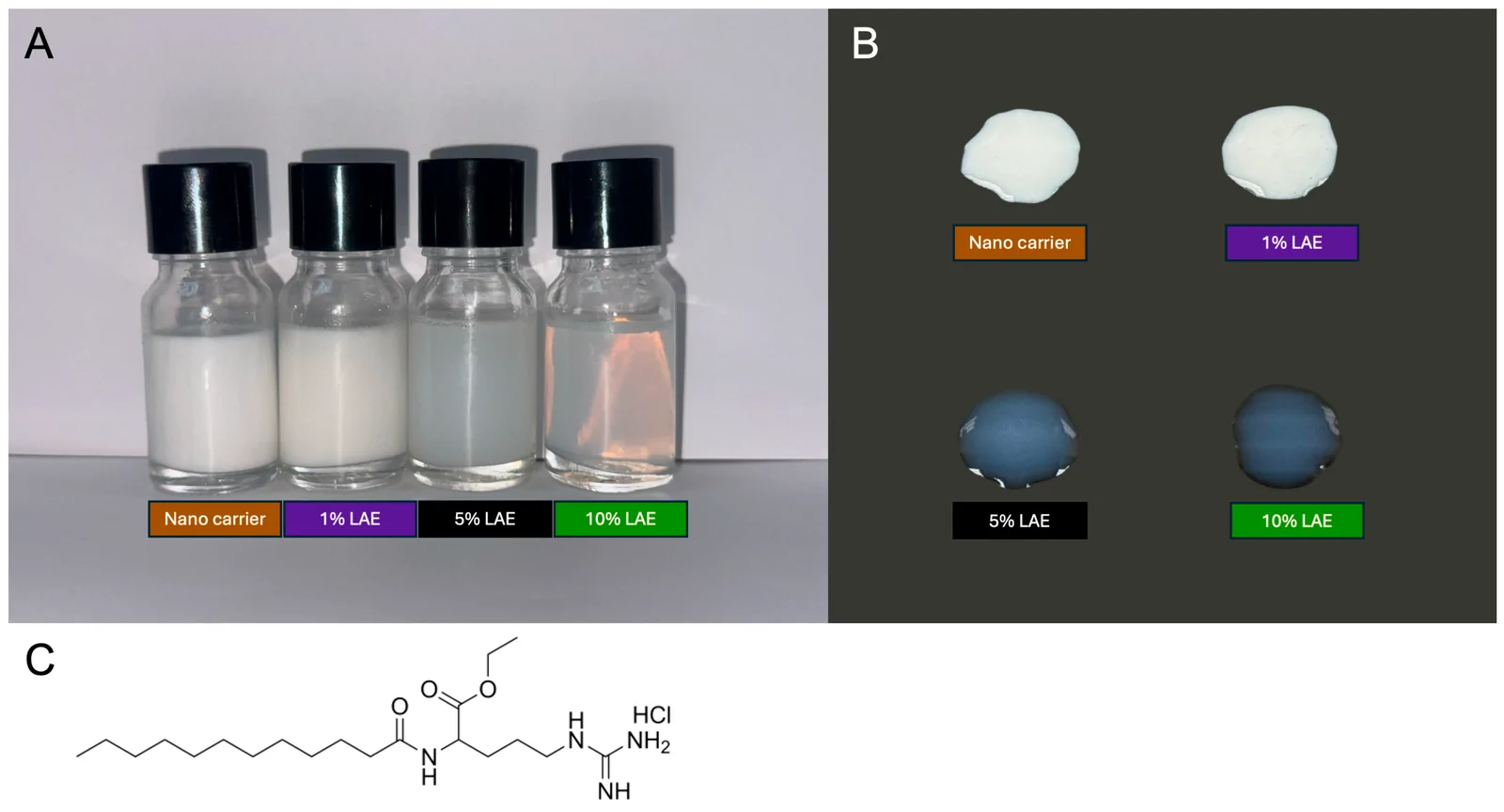
Physicochemical and morphological characterization of LAE-loaded lipid-based nanoparticles (LAE-LBNs). (**A**) Representative photographs showing the macroscopic appearance of the blank nanocarrier and formulations containing increasing concentrations of LAE (1%, 5%, and 10% *w*/*v*). Increasing LAE concentration resulted in a progressive transition from an opaque emulsion to a translucent dispersion. (**B**) Representative photographs of a 30 μL droplet of each formulation dispensed onto a black background to facilitate visual comparison of appearance and opacity. Increasing LAE concentration resulted in a progressive decrease in opacity, with the 5% and 10% LAE formulation appearing more translucent than the blank nanocarrier and 1% LAE formulation. (**C**) Chemical structure of Ethyl Nα-lauroyl-L-arginate hydrochloride (LAE).

The physicochemical characteristics of the developed lipid-based nanoparticles were initially evaluated based on their macroscopic appearance, pH, and morphology. As shown in Figure 1A, the appearance of the nanocarriers was influenced by the LAE concentration. The blank nanocarrier was a milky-white, opaque dispersion, whereas increasing LAE concentrations resulted in progressively greater transparency. The 1% LAE formulation remained largely opaque, while the 5% and 10% LAE formulations appeared translucent and nearly transparent, respectively. Incorporation of LAE also decreased the formulation pH. The pH values were 4.20 ± 0.08 for the blank nanocarrier, 2.85 ± 0.05 for 1% LAE-LBNs, 2.42 ± 0.04 for 5% LAE-LBNs, and 2.18 ± 0.03 for 10% LAE-LBNs. For comparison, the measured pH of 5% LAE-Aq was 2.61 ± 0.08.

### 3.2. Physicochemical Stability of LAE-LBNs

The physicochemical stability of the blank nanocarrier and LAE-containing formulations was evaluated during storage at 4, 25, and 45 °C for two months. Particle size, polydispersity index (PDI), and zeta potential values are summarized in Table 1, and their changes over time are illustrated in Supplementary Figure S1.

The blank nanocarrier exhibited marked increases in particle size and PDI during storage, particularly at 25 and 45 °C, indicating reduced dispersion homogeneity. In comparison, the LAE-containing formulations generally showed smaller changes in their physicochemical properties and retained positive surface charges. However, the 10% LAE formulation developed visible precipitation after one month at 4 °C and showed a reduction in zeta potential during storage.

Among the tested formulations, the 5% LAE formulation demonstrated the most favorable overall physicochemical stability, with a relatively small particle size, narrow size distribution, positive surface charge, and no visible phase separation under the evaluated conditions. Therefore, this formulation was selected for subsequent morphological characterization, encapsulation-efficiency determination, antimicrobial evaluation, and ex vivo follicular-localization studies.

### 3.3. Morphological Characteristics of 5% LAE-LBNs

**Figure 2:**
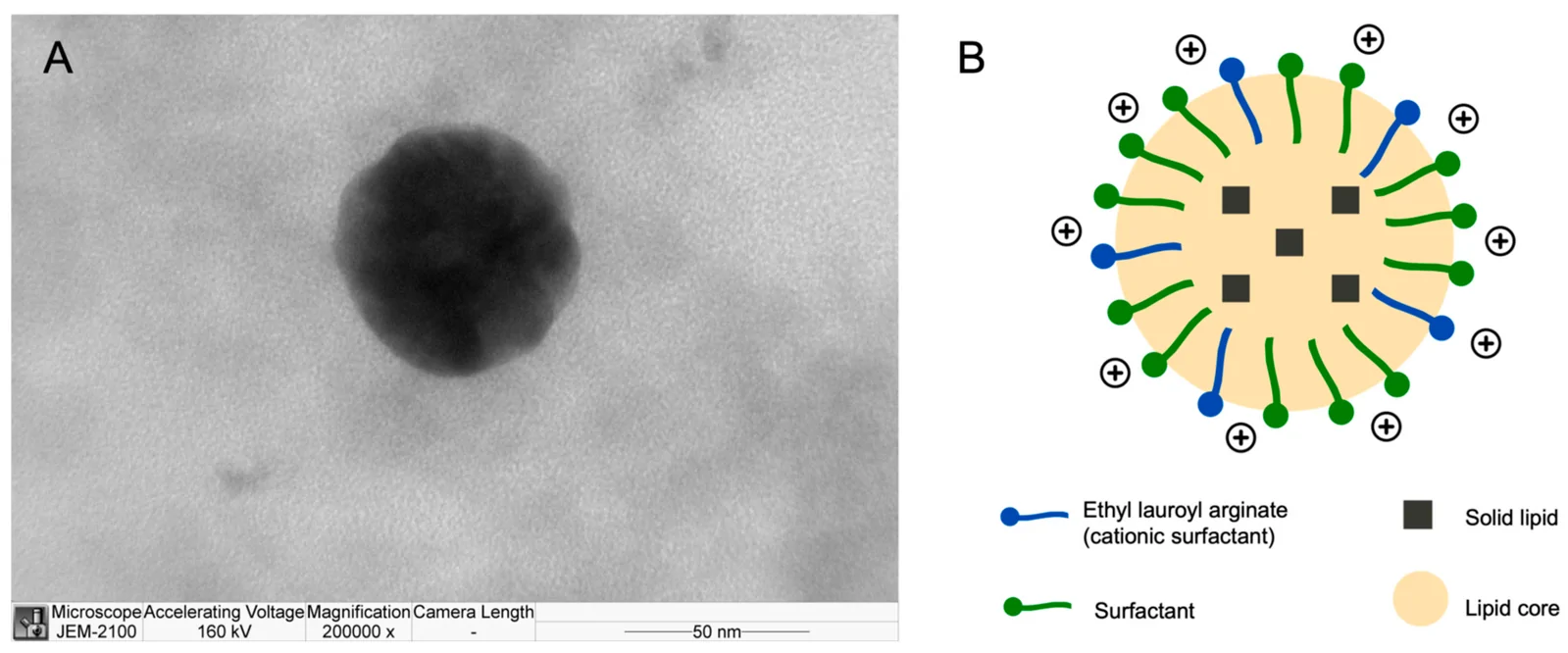
(**A**) Representative transmission electron microscopy (TEM) image of the optimized 5% LAE-LBN formulation, demonstrating uniformly dispersed spherical nanoparticles. Scale bar = 50 nm. (**B**) Schematic illustration of the proposed structure of the LAE-LBNs. The formulation consists of an MCT-based lipid core stabilized by solid lipid, other surfactants, and LAE forming the interfacial layer. The amphiphilic nature of LAE enables its hydrophobic lauroyl chain to partition into the lipid phase while the positively charged arginate head group remains oriented toward the aqueous environment, resulting in a positively charged nanoparticle surface.

Based on its superior initial monodispersity, high thermal stability, and robust cationic charge, the 5% LAE formulation (LAE-LBNs) was selected as the optimized lead candidate for further investigation. The morphological integrity and particle size of this lead formulation were corroborated via Transmission Electron Microscopy (TEM) (Figure 2A). The micrographs revealed well-dispersed, spherical nanoparticles with dimensions consistent with the hydrodynamic diameters obtained through dynamic light scattering (DLS), confirming the successful formation of a stable, discrete nanoparticulate system. A schematic representation of the proposed nanoparticle architecture is shown in Figure 2B.

### 3.4. Fourier-Transform Infrared Spectroscopy

**Figure 3:**
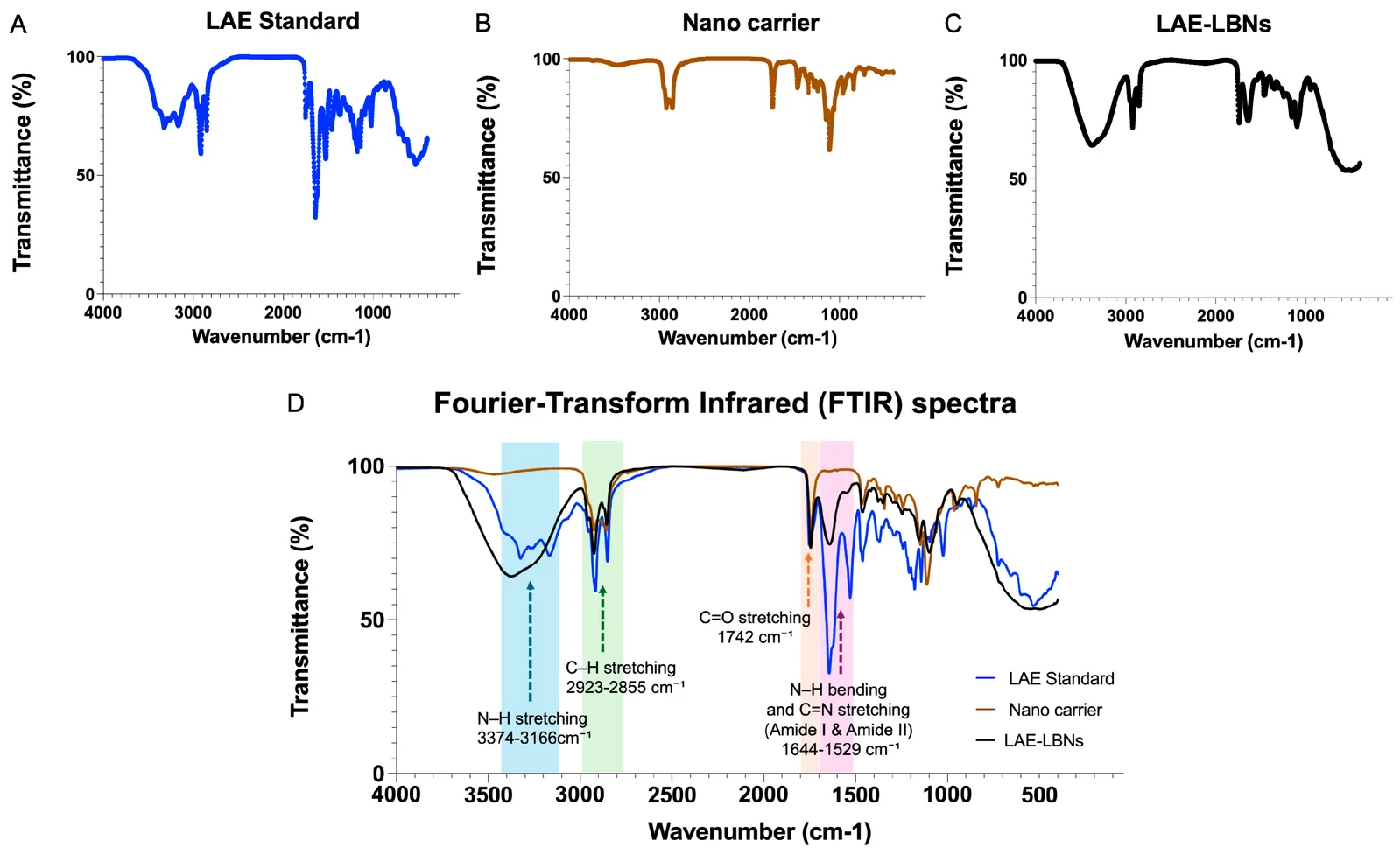
Fourier-transform infrared (FTIR) spectra of (**A**) aqueous LAE used as the reference (LAE-Aq), (**B**) the blank nanocarrier, (**C**) LAE-integrated lipid-based nanoparticles (LAE-LBNs), and (**D**) the overlaid spectra. The blue, brown, and black lines represent LAE-Aq, the blank nanocarrier, and LAE-LBNs, respectively. The shaded regions indicate N–H stretching (3374–3166 cm^−1^), C–H stretching (2923–2855 cm^−1^), ester C=O stretching (1742 cm^−1^), and Amide I and II bands (1644–1529 cm^−1^).

The FTIR spectra of LAE-Aq, the blank nanocarrier, and LAE-LBNs are presented individually in Figure 3A–C, respectively, with the overlaid spectra shown in Figure 3D. The LAE-Aq standard reference spectrum (Figure 3A) exhibited N–H stretching bands at 3324–3166 cm^−1^ and Amide I and II bands at 1644 and 1529 cm^−1^, respectively. These amide bands were absent from the blank nanocarrier spectrum (Figure 3B), which exhibited lipid-associated C–H stretching bands at 2923–2855 cm^−1^ and an ester carbonyl band at 1742 cm^−1^. The LAE-LBN spectrum (Figure 3C) contained bands corresponding to both LAE and the lipid matrix, including N–H stretching at 3374 cm^−1^ and amide bands at 1641 and 1548 cm^−1^. The spectral similarities and differences among the three samples are shown in the overlaid spectra (Figure 3D).

### 3.5. Encapsulation Efficiency of LAE-LBNs

The encapsulation efficiency (EE) of the optimized LAE-LBNs exceeded 99%, indicating that nearly all of the incorporated LAE was retained within the nanoparticle formulation. The drug-loading concentration was fixed at 50 mg/mL (50,000 ppm), selected to match the maximum solubility of LAE in the lipid formulation.

### 3.6. Time-Dependent Appearance of LAE in the Receptor Phase

**Figure 4:**
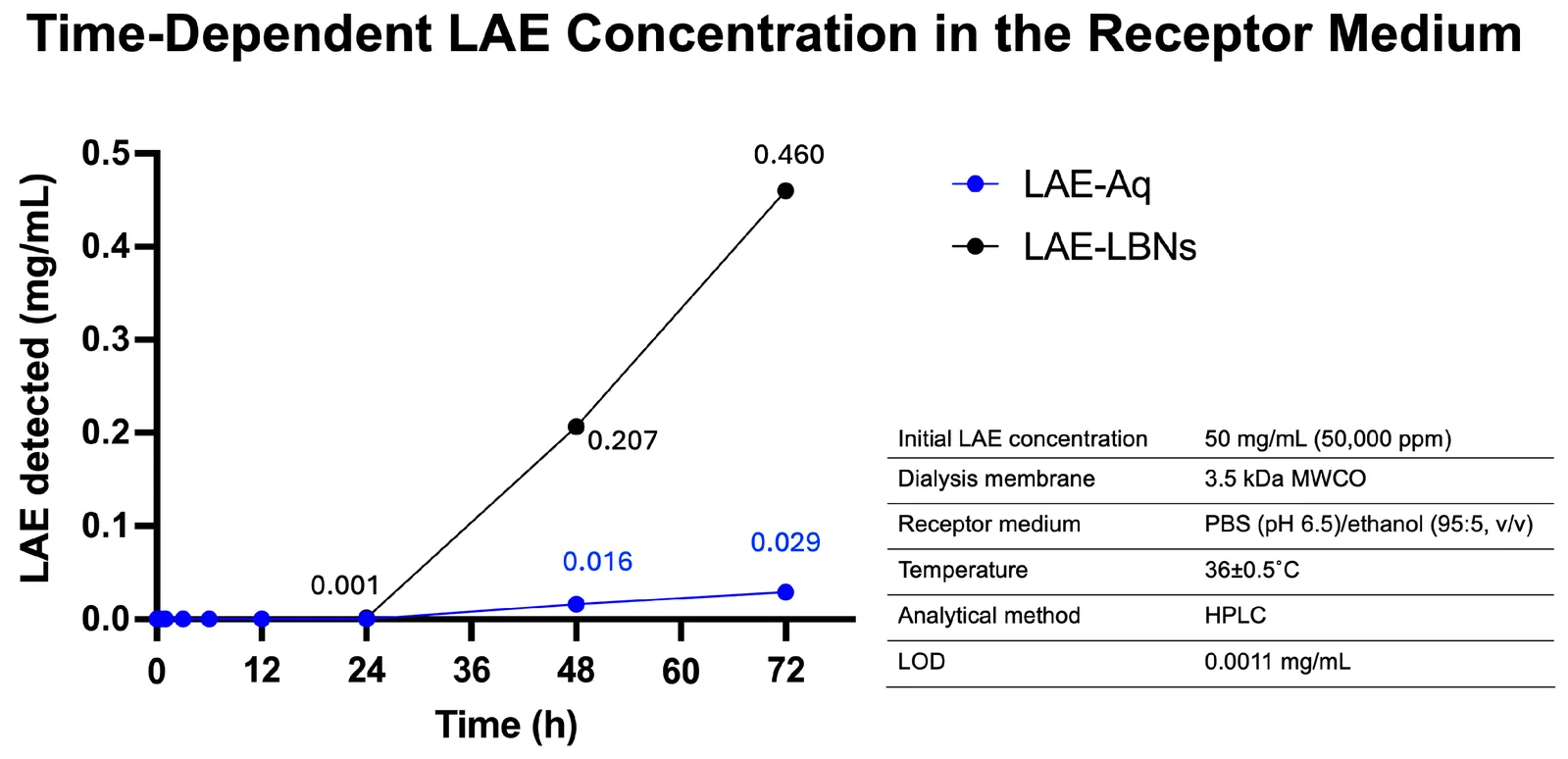
Time-dependent concentrations of LAE detected in the receptor medium following dialysis of aqueous LAE (LAE-Aq) and LAE-loaded lipid-based nanoparticles (LAE-LBNs) in PBS (pH 6.5)/ethanol (95:5, *v*/*v*) at 36 °C. LAE concentrations were determined by HPLC over 72 h. Concentrations below the analytical limit of detection (LOD; 0.0011 mg/mL) are indicated as <LOD. Lines connecting the data points are included as visual guides only. The measured concentrations represent LAE detected in the receptor medium under the specified dialysis conditions and were not corrected for potential adsorption to the dialysis membrane or nylon syringe filter.

The concentrations of LAE detected in the receptor phase following dialysis of LAE-LBNs and LAE-Aq are presented in Figure 4. Both formulations contained an initial LAE concentration of 5% (50 mg/mL). For LAE-LBNs, receptor-phase LAE concentrations remained below the analytical limit of detection (LOD; 0.0011 mg/mL) during the first 12 h. At 24 h, LAE was detected at approximately the LOD (0.0011 mg/mL), followed by an increase to 0.2065 mg/mL at 48 h and 0.4600 mg/mL at 72 h. For LAE-Aq, LAE remained below the LOD through 24 h, after which concentrations of 0.0160 and 0.0288 mg/mL were detected at 48 and 72 h, respectively. Under the same dialysis conditions, the LAE concentration detected in the receptor phase at 72 h was approximately 16-fold higher for LAE-LBNs than for LAE-Aq.

### 3.7. Antimicrobial Activity of LAE-LBNs

The antimicrobial activity of the blank nanocarrier, 5% LAE-LBNs, and 5% aqueous LAE (LAE-Aq) was evaluated against three clinically relevant canine skin pathogens: *Malassezia pachydermatis*, methicillin-resistant *Staphylococcus pseudintermedius* (MRSP), and methicillin-susceptible *S. pseudintermedius* (MSSP).

The blank nanocarrier exhibited no detectable bactericidal or fungicidal activity against any of the tested microorganisms at the concentrations evaluated. In contrast, 5% LAE-LBNs and 5% LAE-Aq produced identical antimicrobial endpoints. The MBCs against MRSP and MSSP and the MFC against *M. pachydermatis* were all observed at a 1:4096 dilution. Based on an initial LAE concentration of 50 mg/mL (50,000 ppm), this dilution corresponded to an LAE concentration of approximately 0.0122 mg/mL (12.2 µg/mL or approximately 12.2 ppm). No microbial growth was detected upon subculture after 1 h of exposure at the corresponding MBC or MFC.

All replicate endpoint determinations were concordant, yielding the same MBC or MFC of 0.0122 mg/mL. Because these endpoints were determined using twofold serial dilutions and therefore represent discrete dilution-based measurements, repeatability is reported as endpoint concordance rather than as mean ± standard deviation.

### 3.8. Ex Vivo Skin Localization of LAE-LBNs

**Figure 5:**
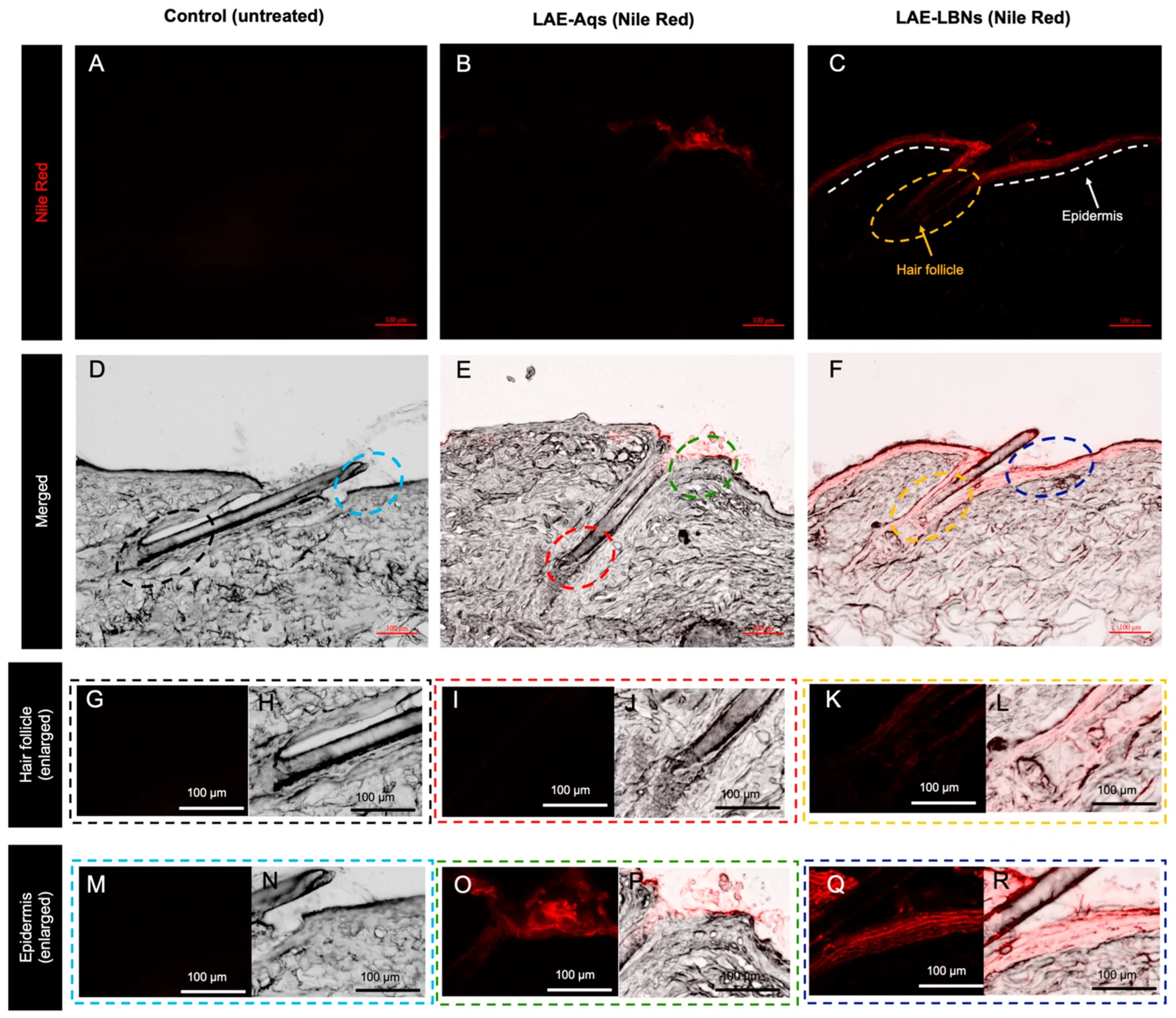
Ex vivo localization of Nile Red-labeled LAE-loaded lipid-based nanoparticles (LAE-LBNs) in porcine ear skin following topical application. (**A**–**C**) Representative fluorescence and (**D**–**F**) merged fluorescence/brightfield images of skin treated with (**A**,**D**) untreated control skin, (**B**,**E**) LAE-Aq, and (**C**,**F**) LAE-LBNs. Fluorescence signals were observed predominantly within the stratum corneum, epidermis, and hair follicle in LAE-LBNs-treated skin. Colored dashed circles in panels (**D**–**F**) correspond to the enlarged regions below. Panels (**G**–**R**) show enlarged views of the hair follicle region from (**G**,**H**) untreated skin, (**I**,**J**) LAE-Aq, and (**K**,**L**) skin treated with LAE-LBNs. Panels (**M**–**R**) present enlarged views of the epidermal region from (**M**,**N**) untreated skin, (**O**,**P**) LAE-Aq, and (**Q**,**R**) skin treated with LAE-LBNs. Scale bars = 100 µm.

Representative fluorescence microscopy images of porcine ear skin following topical application of Nile Red-labelled formulations are shown in Figure 5. Untreated control skin exhibited negligible background fluorescence (Figure 5A,D,G,H,M,N). In contrast, skin treated with Nile Red-labelled LAE-Aq showed fluorescence primarily confined to the stratum corneum, with little or no detectable signal in the underlying epidermis or hair follicle (Figure 5B,E,I,J,O,P). LAE-LBN-treated skin displayed substantially greater fluorescence intensity, with prominent localization throughout the stratum corneum and viable epidermis, together with a distinct fluorescent signal extending along the hair follicle (Figure 5C,F,K,L,Q,R). The merged fluorescence and brightfield images further confirmed the localization of the fluorescent signal within both the epidermal and follicular compartments.

Higher-magnification images of the hair follicle (Figure 5G–L) demonstrated that fluorescence was absent in untreated skin and minimal following LAE-Aq application, whereas LAE-LBNs produced pronounced fluorescence surrounding and extending along the follicular canal. Similarly, enlarged views of the epidermis (Figure 5M–R) showed that LAE-Aq fluorescence remained largely restricted to the superficial stratum corneum, while LAE-LBNs exhibited more extensive fluorescence throughout the epidermis.

## 4. Discussion

LAE-integrated lipid-based nanoparticles (LAE-LBNs) were successfully developed as stable oil-in-water (O/W) nanosystems using a combination of high-shear homogenization and probe ultrasonication. High-energy emulsification is widely employed for the preparation of lipid nanocarriers because high-shear homogenization produces fine emulsion droplets, while ultrasonication further reduces droplet size through acoustic cavitation, yielding nanoparticles with narrow size distributions and high colloidal stability [36,37]. Compared with high-pressure homogenization, this solvent-free approach is simple, reproducible, readily scalable, and well suited for laboratory-scale development and industrial manufacture of topical lipid nanocarriers [38,39]. Moreover, compared with low-energy emulsification techniques, high-energy emulsification generally requires lower surfactant concentrations while maintaining kinetically stable nanosystems, making it an attractive manufacturing strategy for pharmaceutical applications [40,41].

A distinctive feature of the present formulation is the multifunctional role of LAE within the nanoparticle architecture. Unlike conventional lipid nanocarriers, in which the active ingredient and stabilizing excipients perform separate functions, LAE simultaneously acts as the antimicrobial agent, cationic surface modifier, and interfacial stabilizer owing to its amphiphilic molecular structure [17]. During emulsification, LAE is expected to preferentially adsorb at the oil-water interface, orienting its hydrophobic lauroyl chain toward the MCT-lipid core while exposing its positively charged arginate head group to the aqueous phase [24,42]. This molecular arrangement lowers interfacial tension, facilitates droplet disruption during emulsification, and generates a highly cationic nanoparticle surface [41]. Consequently, LAE becomes an integral structural component of the nanocarrier, which helps simplify formulation design while simultaneously contributing to colloidal stability and antimicrobial functionality.

The excellent physicochemical stability of the optimized formulation can be attributed to its balanced interfacial composition. The calculated hydrophilic–lipophilic balance (HLB) of 8.90, derived from Span 80 (HLB 4.3), LAE (HLB 10.5), and PEG-100 stearate (HLB 18.8), falls within the optimal range for stabilizing O/W emulsions (HLB 8-18) [15,43,44]. Considering a surfactant-to-oil ratio of 53.5%, this composition likely promoted efficient interfacial coverage and complementary packing of surfactant molecules around the lipid droplets. Furthermore, cetyl alcohol and cholesterol likely reinforced the interfacial film by increasing its rigidity and reducing molecular mobility, thereby suppressing droplet coalescence and Ostwald ripening, a destabilization process in which smaller droplets dissolve and diffuse toward larger droplets because of differences in Laplace pressure and chemical potential, ultimately leading to particle growth during storage [45,46,47].

Consistent with these design characteristics, the 5% LAE-LBN formulation exhibited the most favorable physicochemical properties, including a small particle size, narrow size distribution, high positive zeta potential, and excellent long-term stability under refrigerated, ambient, and accelerated storage conditions. Stability across this temperature range demonstrates the robustness of the interfacial architecture under conditions relevant to product transportation and storage. Elevated temperatures generally accelerate droplet diffusion, surfactant desorption, and Ostwald ripening, whereas low temperatures may induce lipid crystallization or polymorphic transitions that compromise colloidal stability [8,41,48]. The stability of the optimized formulation is likely attributed to the combined effects of electrostatic and steric stabilization from the highly positive zeta potential, which generated sufficient electrostatic repulsion between nanoparticles, while PEG-containing surfactants limited particle aggregation through steric hindrance [49]. In contrast, increasing the LAE concentration to 10% reduced formulation stability, resulting in phase separation during storage. This finding suggests that excessive LAE saturated the available interfacial area and increased ionic strength, thereby weakening electrostatic repulsion and promoting aggregation in accordance with DLVO theory [15,24,50,51]. These findings indicate that long-term stability depended on achieving an optimal balance between interfacial composition and particle interactions rather than simply increasing the surfactant concentration.

An important consideration for topical application is the acidic pH of the optimized 5% LAE-LBN formulation (2.42 ± 0.04), which was slightly lower than that of 5% LAE-Aq (pH 2.61) and below the reported pH range of intact adult canine skin (4.40–8.18) [52]. Although repeated application of highly acidic formulations may increase the risk of local irritation, particularly on compromised skin, the acidic environment may also provide an additional antimicrobial advantage. Previous studies have shown that *Malassezia pachydermatis* growth is inhibited at pH values below 4.0 [53]. Therefore, the acidic pH may complement the membrane-disruptive activity of amphiphilic cationic compounds such as LAE and contribute to the potent fungicidal activity observed against *M. pachydermatis* [53,54]. Nevertheless, future studies should evaluate cutaneous tolerability following repeated topical administration, and formulation optimization through buffering systems or pH-adjusting excipients may improve skin compatibility while preserving nanoparticle stability and antimicrobial efficacy.

FTIR provided qualitative confirmation of LAE-associated bands in the LAE-LBN formulation. The characteristic N–H stretching and amide-related bands observed in LAE-Aq were also detected in LAE-LBNs but were absent from the blank nanocarrier. Because the LAE-LBN spectrum contained overlapping contributions from LAE and the lipid excipients, FTIR was used only to support the presence of LAE and not to establish molecular interactions, encapsulation, or chemical stability. Encapsulation was evaluated separately by HPLC, which demonstrated an encapsulation efficiency exceeding 99%. The functional performance of the formulation was subsequently evaluated using complementary dialysis-based transfer and direct antimicrobial assessments.

The dialysis assessment revealed a distinct time-dependent LAE transfer pattern. Receptor-phase LAE remained below the analytical limit of detection during the first 12 h, became detectable after 24 h, and increased further at 48 and 72 h. At 72 h, the LAE concentration detected for LAE-LBNs was approximately 16-fold higher than that for LAE-Aq under identical conditions. The progressive appearance of LAE in the receptor medium suggests formulation-dependent transfer under the evaluated conditions. However, receptor-phase dialysis profiles can be governed partly by transport across the membrane and may therefore not directly represent intrinsic nanoparticle release kinetics [55]. Moreover, the observed profile may depend on factors such as drug concentration, solubility, formulation composition, and dialysis configuration [56]. Accordingly, the present results are interpreted as a descriptive measure of formulation performance rather than as definitive release kinetics.

Complementing these findings, LAE-LBNs exhibited rapid bactericidal and fungicidal activity against MSSP, MRSP, and *M. pachydermatis* after 1 h of direct exposure, with MBC/MFC values identical to those of LAE-Aq. The blank nanocarrier showed no detectable activity, confirming that LAE was primarily responsible for the observed antimicrobial effect. This rapid activity is consistent with the reported ability of LAE to alter microbial membrane potential and permeability [18]. Together, the two assessments demonstrate complementary aspects of formulation performance: preservation of rapid direct-contact antimicrobial activity and time-dependent transfer of LAE into the surrounding medium from 24 h onward. These findings support the functional performance of LAE-LBNs without assuming a direct quantitative relationship between the antimicrobial and dialysis experiments.

For comparison, Sayem et al. [57] reported amoxicillin MBC values of 0.125–128 µg/mL against canine *S. pseudintermedius* isolates, with MBC_50_ and MBC_90_ values of 1 and 8 µg/mL, respectively. The LAE-LBN MBC observed in the present study (12.2 µg/mL) was within this range but higher than the reported amoxicillin MBC_90_. For canine *M. pachydermatis* isolates, Jerzsele et al. [58] reported MFC_90_ values of 0.125 and 0.25 µg/mL for ketoconazole and itraconazole, respectively. These values were substantially lower than the MFC of LAE-LBNs (12.2 µg/mL), indicating greater in vitro antifungal potency of the azoles on a mass-concentration basis. However, LAE demonstrated both bactericidal and fungicidal activity in the present study, whereas ketoconazole and itraconazole are specifically antifungal agents. Such broad-spectrum activity may be clinically relevant because bacterial and yeast involvement can occur concurrently in canine skin disease. For example, Chansiripornchai and Sukanan [59] reported an atopic dog with concurrent *M. pachydermatis* and coccoid bacterial populations on skin cytology, requiring both antifungal and antibacterial therapy. Thus, a single topical platform active against both *S. pseudintermedius* and *M. pachydermatis* may offer a useful strategy for managing canine skin conditions in which these microorganisms coexist.

Ex vivo fluorescence imaging further demonstrated preferential localization of Nile Red-labeled LAE-LBNs within the epidermis and hair follicles following topical application. Hair follicles are recognized as important penetration pathways and drug reservoirs for nanoparticle-based topical delivery systems, particularly in densely haired skin, where they facilitate prolonged retention of topically applied formulations [60,61]. This localization is particularly relevant because hair follicles and associated pilosebaceous units serve as major ecological niches for *Staphylococcus pseudintermedius* and *Malassezia pachydermatis*, contributing to persistent microbial colonization and recurrent superficial skin infections [4,62,63,64,65]. Consequently, preferential follicular accumulation may position LAE-LBNs within clinically relevant sites of microbial colonization and promote local retention of the nanocarriers within these skin structures. In this study, porcine skin was selected as a representative ex vivo model because of its established suitability for evaluating mammalian skin permeability and topical delivery performance. While this standardized model enabled mechanistic investigation of nanoparticle-skin interactions, further studies using canine skin and clinically relevant infection models are required to confirm follicular delivery, skin retention, and therapeutic efficacy under physiological conditions.

LAE-LBNs did not alter the inherent bactericidal or fungicidal activity of LAE. Instead, nanoparticle incorporation provided additional formulation-related advantages, including improved physicochemical stability and preferential localization within clinically relevant skin structures [66,67]. These characteristics are particularly relevant to veterinary dermatology, where conventional topical formulations may be removed through grooming, bathing, or mechanical abrasion, potentially requiring repeated application and reducing owner compliance [68].

## 5. Conclusions

This study developed ethyl lauroyl arginate-integrated lipid-based nanoparticles (LAE-LBNs) as a multifunctional non-antibiotic topical delivery platform. The optimized 5% LAE-LBN formulation exhibited favorable physicochemical stability, encapsulation efficiency exceeding 99%, and a strongly positive surface charge. Dialysis assessment demonstrated time-dependent transfer of LAE into the receptor medium, with LAE becoming detectable from 24 h onward. In direct antimicrobial assays, LAE-LBNs exhibited bactericidal and fungicidal activity against methicillin-resistant and methicillin-susceptible *Staphylococcus pseudintermedius* and *Malassezia pachydermatis* after 1 h of exposure. The identical MBC/MFC values (12.2 µg/mL) obtained for LAE-LBNs and LAE-Aq indicated that nanoparticle incorporation preserved the antimicrobial activity of LAE. Furthermore, fluorescence associated with the labeled LAE-LBN formulation was observed within the stratum corneum, viable epidermis, and hair follicles, whereas the aqueous formulation was predominantly confined to the stratum corneum.

These findings demonstrate complementary aspects of LAE-LBN performance, including physicochemical stability, rapid direct-contact antimicrobial activity, time-dependent LAE transfer, and localization within clinically relevant skin structures. The results support further investigation of LAE-LBNs as a non-antibiotic topical delivery platform for bacterial- and yeast-associated canine skin infections. Future studies should include membrane and filter recovery, complete LAE mass-balance analysis, quantitative cutaneous-retention assessment, repeated-dose skin-safety evaluation, and confirmation of therapeutic efficacy using canine skin and clinically relevant in vivo infection models.

## Figures and Tables

**Table 1:** Physicochemical stability of the blank nanocarrier, 1%, 5%, and 10% LAE lipid-based nanoparticles under different storage conditions.

Formulation	Storage Temperature	Time	Particle Size (nm)	PDI	Zeta Potential (mV)
Blank nanocarrier		Day 0	202.20 ± 2.72	0.20 ± 0.01	−1.64 ± 1.55
	4 °C	1 month	224.50 ± 6.30	0.28 ± 0.02	−7.38 ± 0.99
		2 months	259.00 ± 8.06	0.38 ± 0.05	−22.02 ± 0.39
	25 °C	1 month	238.87 ± 2.64	0.25 ± 0.02	−6.16 ± 0.43
		2 months	438.17 ± 11.87	0.52 ± 0.1	−17.12 ± 0.63
	45 °C	1 month	540.77 ± 16.58	0.51 ± 0.02	−11.38 ± 1.64
		2 months	775.53 ± 31.02	0.54 ± 0.02	−28.99 ± 1.20
1% LAE-		Day 0	131.23 ± 0.95	0.19 ± 0.01	23.28 ± 2.58
	4 °C	1 month	139.30 ± 1.45	0.23 ± 0.02	26.80 ± 2.58
		2 months	143.27 ± 5.41	0.26 ± 0.17	32.69 ± 1.85
	25 °C	1 month	131.57 ± 3.30	0.21 ± 0.01	28.06 ± 0.89
		2 months	165.10 ± 4.18	0.32 ± 0.03	32.98 ± 1.03
	45 °C	1 month	146.77 ± 1.31	0.18 ± 0.02	35.47 ± 1.88
		2 months	149.33 ± 4.29	0.22 ± 0.01	41.11 ± 0.82
5% LAE		Day 0	61.46 ± 0.55	0.06 ± 0.02	46.97 ± 10.80
	4 °C	1 month	93.41 ± 0.33	0.24 ± 0.01	47.04 ± 2.72
		2 months	158.00 ± 0.60	0.35 ± 0.01	44.89 ± 3.65
	25 °C	1 month	62.86 ± 1.50	0.08 ± 0.02	31.42 ± 7.97
		2 months	84.98 ± 1.23	0.10 ± 0.02	32.60 ± 2.27
	45 °C	1 month	91.07 ± 1.36	0.10 ± 0.02	34.45 ± 1.76
		2 months	114.70 ± 0.36	0.27 ± 0.02	48.65 ± 4.46
10% LAE		Day 0	61.28 ± 0.43	0.41 ± 0.003	30.36 ± 1.62
	4 °C	1 month	Phase separation (precipitated)		
		2 months	Phase separation (precipitated)		
	25 °C	1 month	76.08 ± 3.02	0.24 ± 0.02	37.65 ± 9.08
		2 months	96.62 ± 3.79	0.27 ± 0.01	7.94 ± 1.55
	45 °C	1 month	83.06 ± 1.01	0.07 ± 0.03	39.99 ± 0.76
		2 months	99.22 ± 1.29	0.05 ± 0.04	18.96 ± 1.47

## Data Availability

The original contributions presented in this study are included in the article. Further inquiries can be directed to the corresponding authors.

## Supplementary Materials

The following supporting information can be downloaded at: https://www.mdpi.com/article/10.3390/pharmaceutics18091165/s1, Table S1: pH of nanocarrier, LAE-LBNs, and LAE-Aq, Figure S1: Physicochemical stability of nanocarriers and lipid-based nanoparticles at different LAE concentrations (1%, 5%, and 10%) during 2 months of storage at 4, 25, and 45 °C.

## Abbreviations

The following abbreviations are used in this manuscript:

CFU	Colony-forming units
CSP	Canine superficial pyoderma
DLS	Dynamic light scattering
DLVO	Derjaguin–Landau–Verwey–Overbeek
EE	Encapsulation efficiency
FT-IR	Fourier transform infrared
HLB	Hydrophilic-lipophilic balance
HPLC	High-performance liquid chromatography
LAE	Ethyl Nα-lauroyl-L-arginate hydrochloride
LAE-Aq	Ethyl lauroyl arginate aqueous solution
LAE-LBNs	Ethyl lauroyl arginate-integrated lipid-based nanoparticles
LBNs	Lipid-based nanoparticles
MALDI-TOF MS	Matrix-assisted laser desorption/ionization time-of-flight mass spectrometry
MBC	Minimum bactericidal concentration
MCT	Medium chain triglyceride
MFC	Minimum fungicidal concentration
MRSP	Methicillin-resistant *Staphylococcus pseudintermedius*
MSSP	Methicillin-susceptible *Staphylococcus pseudintermedius*
MWCO	Molecular weight cut-off
O/W	Oil-in-water
PEG	Polyethylene glycol
PDI	Polydispersity index
PBS	Phosphate-buffered saline
SDA	Sabouraud dextrose agar
Span 80	sorbitan monooleate
TEM	Transmission electron microscopy
