# Supplementary material for: Ethyl Lauroyl Arginate-Integrated Lipid Nanoparticles as a Multifunctional Non-Antibiotic Platform: Physicochemical Stability, Follicular Penetration, and Broad-Spectrum Activity Against Canine Skin Pathogens
Source: Pharmaceutics. 2026 Sep 16;18(9):1165. doi: 10.3390/pharmaceutics18091165 (PMC13609942; doi:10.3390/pharmaceutics18091165)
Supplement: Supplementary file 1 [file pharmaceutics-18-01165-s001.zip › pharmaceutics-4489296-supplementary.pdf]

Supplement Table

Table S1. pH of nanocarrier, LAE-LBNs, and LAE-Aq

Formulation	pH	Interpretation
Nanocarrier (no LAE)	4.20 ± 0.08	Weakly acidic
1% LAE-LBNs	2.85 ± 0.05	Strongly acidic
5% LAE-LBNs	2.42 ± 0.04	More acidic than 1%
10% LAE-LBNs	2.18 ± 0.03	Most acidic
5% LAE-Aq	2.61 ± 0.08.	Relatively similar to 5%LAE-LBNs

Supplementary figure caption

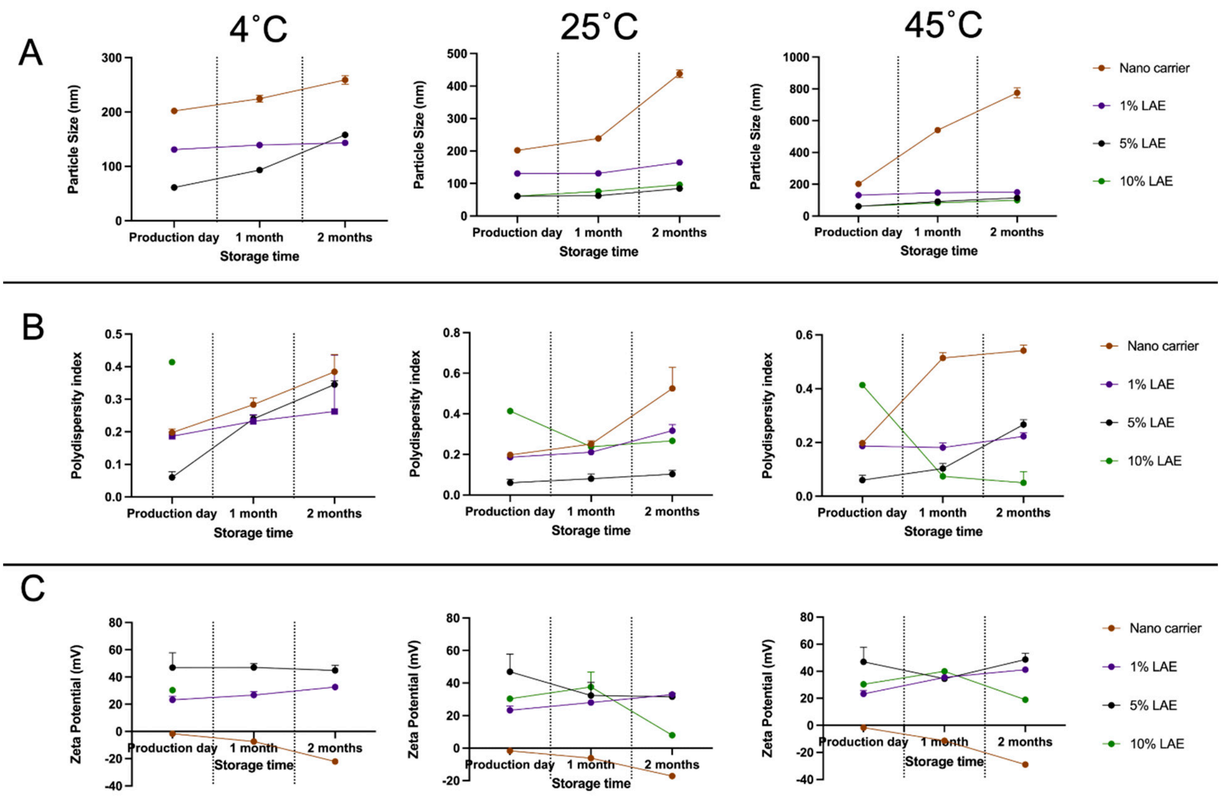

Figure S1. Physicochemical stability of nanocarriers and lipid-based nanoparticles at different LAE concentrations (1%, 5%, and 10%) during 2 months of storage at 4, 25, and 45 °C. (**A**) Particle size (nm), (**B**) polydispersity index (PDI), and (**C**) zeta potential (mV). Data are presented as mean ± SD (n = 3).
